# Opportunities to improve policy dissemination by tailoring communication materials to the research priorities of legislators

**DOI:** 10.1186/s43058-022-00274-6

**Published:** 2022-03-04

**Authors:** Natalie R. Smith, Stephanie Mazzucca, Marissa G. Hall, Kristen Hassmiller Lich, Ross C. Brownson, Leah Frerichs

**Affiliations:** 1 Department of Social and Behavioral Sciences, Harvard TH Chan School of Public Health, 677 Huntington Avenue, SBS 7th floor, MA 02115 Boston, USA; 2 Prevention Research Center, Brown School, Washington University in St. Louis, Saint Louis, USA; 3 Department of Health Behavior, Gillings School of Global Public Health, UNC Chapel Hill, Chapel Hill, USA; 4 Lineberger Comprehensive Cancer Center, UNC Chapel Hill, Chapel Hill, USA; 5 Carolina Population Center, UNC Chapel Hill, Chapel Hill, USA; 6 Department of Health Policy and Management, Gillings School of Global Public Health, UNC Chapel Hill, Chapel Hill, USA; 7 Department of Surgery (Division of Public Health Sciences) and Alvin J Siteman Cancer Center, Washington University School of Medicine, Washington University in St. Louis, Saint Louis, USA; 8 Department of Health Policy and Management, Gillings School of Global Public Health, UNC Chapel Hill, Chapel Hill, USA

**Keywords:** Information dissemination, Health policy, Policy making, Implementation science

## Abstract

**Background:**

Communicating research to policymakers is a complex and difficult process. Ensuring that communication materials have information or design aspects that appeal to groups of policymakers with different priorities could be a substantive improvement over current dissemination approaches. To facilitate a more nuanced design of policy communication materials and message framing, we identified and characterized groups of state legislators based on how they prioritize different characteristics of research.

**Methods:**

We used deidentified data collected in 2012 on 862 state legislators belonging to the US liberal-moderate-conservative ideological spectrum and from all 50 US states. Legislators were grouped using latent class analysis based on how they prioritized 12 different characteristics of research (e.g., research is unbiased, presents data on cost-effectiveness, policy options are feasible). We fit initial models using 1–6 group solutions and chose the final model based on identification, information criteria, and substantive interpretation.

**Results:**

Most legislators placed a high priority on research that was understandable (61%), unbiased (61%), available at the time that decisions are made (58%), and brief and concise (55%). The best model identified four groups of state legislators. Pragmatic consumers (36%) prioritized research that was brief and concise, provided cost-effectiveness analyses, and was understandably written. Uninterested skeptics (30%) generally did not place a high priority on any of the research characteristics. Conversely, one-quarter of legislators (25%) belonged to the Highly Informed Supporters group that placed a high priority on most characteristics of research. Finally, Constituent-Oriented Decision Makers (9%) prioritized research that was relevant to their constituents, delivered by someone they knew or trusted, available at the time decisions were made, and dealt with an issue that they felt was a priority for state legislative action.

**Conclusions:**

To maximize the impact of dissemination efforts, researchers should consider how to communicate with legislators who have distinct preferences, values, and priorities. The groups identified in this study could be used to develop communication materials that appeal to a wide range of legislators with distinct needs and preferences, potentially improving the uptake of research into the policymaking process. Future work should investigate how to engage skeptical legislators.

**Supplementary Information:**

The online version contains supplementary material available at 10.1186/s43058-022-00274-6.

Contributions to the literature

- Incorporating research into public health policymaking can promote the efficient use of limited resources and improve policy implementation; however, effective communication of research to policy audiences is difficult.
- Research has shown that effective communication depends on a variety of factors, including how research is presented to policymakers. We build on this work by investigating shared preferences for research presentation among groups of state legislators.
- Four distinct groups of shared preferences emerged in our work, which researchers can use to better communicate their work to policy audiences.

## Background

Evidence-informed policies are critical for addressing a wide array of public health challenges [1]. Major public health milestones in the USA have been achieved thanks to policies such as mandatory blood donor screening, a federal cigarette excise tax, mandatory folic acid fortification of cereal, and seat belt laws. Indeed, using evidence to inform policymaking is a key principle for effective public health policy change [1] and can promote the uptake of effective policies and programs, efficient use of resources, and more effective implementation [2]. However, policies, particularly in the USA, are commonly enacted based on short-term opportunities or crises, lacking systematic attention to research evidence [2–6]. For example, a study focusing on Minnesota obesity-related legislation found that only 41% of legislative materials mentioned evidence, while 92% of legislative materials included non-research information such as constituent opinion or anecdotes [6]. How research is packaged is also critical. For example, it is often helpful to weave complex, scientific evidence into stories that will resonate with policy makers [7].

Broadly, there are many factors that influence policymaking processes, including context and opportunity, but the importance of how research is used and framed is a common component of many policymaking theories and frameworks [8]. Numerous studies have examined factors that prevent research from being used in the policymaking process [9–11]. In general, we know that a wide variety of characteristics influence the likelihood of research being used in policymaking—including barriers like a lack of personal contact between researchers and decision makers [9–11], managing competing influences on policymaking [9], and timeliness of research findings [10, 11]. Broadly, these barriers can be bucketed into general themes of organizations and resources, policymaker characteristics, policy characteristics, research, and researcher characteristics [10]. This final theme, research and researcher characteristics, is most directly modifiable by researchers interested in improving the use of research in policymaking, and studies have suggested that improved, dedicated dissemination efforts are critical [10].

Recent work has indicated a variety of potential strategies that researchers could use to improve dissemination efforts, including engaging policymakers early, understanding context, knowing the policy players, and making sure research products are timely, relevant, and accessible [12]. Experimental studies have also been used to identify key dissemination insights. In the USA, Brownson et al. compared multiple policy briefs with varied introductory framing (data vs. stories) and focus (state vs. local) [13]. Their work showed that policymakers had substantially different preferences for the briefs based on their policymaking position (legislator vs. staffer or executive), age, education, political party, and ideologies [13]. Preferences were different both between and within groups—for example, staffers and legislators differed in their preferences, but there was also variation within staffers [13].

This previous research clarifies that communicating to policymakers with a one-size-fits-all approach will likely be ineffective, and research dissemination materials should include elements that are tailored to different policymakers [12–14]. However, there are still outstanding questions about how to tailor. Policy communication materials could be tailored to individuals, but this may not be efficient. An alternative approach is to ensure that policy briefs are designed with a heterogeneous audience in mind. This approach can be informed using audience segmentation methods. Audience segmentation focuses on segmenting a large population into groups with similar needs and preferences, while acknowledging that there will always be heterogeneity between individuals [15–17].

If specific groups of legislators with cohesive research preferences can be identified, then knowledge of those preferences could be incorporated into policy briefs, increasing the likelihood that a broader range of policymakers will find them accessible and useful. Recent work has used data-driven methods such as latent class analysis (LCA) to identify audience segments of state legislators based on their personal stances on behavioral health issues [16]. Ideally, we also need to understand how legislators can be grouped based on their research information preferences, without restricting to a specific content area. In this paper, we aim to use LCA to identify whether legislators can be grouped based on stated preferences for research information.

## Methods

We used data collected in 2012 from 862 US state legislators. Legislators were randomly sampled from a list provided by the National Conference of State Legislators [18, 19]. A total of 1880 legislators were contacted up to 10 times; 862 agreed to participate (46% response rate) [18, 19]. Legislators were asked a variety of open- and closed-ended questions that were adapted from previous studies of state legislators [20, 21]. These questions covered basic demographic and political information, top health issues of interest, sources of information, and preferences for research (full questionnaire in Additional file 1).

Legislators were grouped into mutually exclusive and exhaustive audience segments using LCA. LCA assumes that there are certain latent constructs (classes) that cannot be directly measured by researchers [22]. LCA models estimate classes by using observed categorical variables related to class membership while accounting for possible measurement error in those observed variables [22]. We used 12 indicator variables of research preferences (e.g., information is unbiased) that were originally measured on a scale of 1 to 5, with 1 meaning low priority and 5 meaning high priority. These variables were dichotomized for use in the LCA (=1 if high priority, 0 otherwise) to aid interpretations of the final audience segments. To select the final LCA model we first fit models using 1–6 class solutions, each using full-information maximum likelihood estimation methods and 1000 random starting values to assess model convergence. Using full information maximum likelihood allows for item-wise missing data within observations; only observations with missing data on all 12 LCA inputs were dropped (*n*=2). We chose our final model based on model identification, information criteria statistics, and substantive interpretation [16, 22].

We output predicted probabilities of class membership for each legislator and assigned each legislator to the class for which they had the highest probability of belonging. We display descriptive statistics within each class focusing on demographic, political, research-related, and legislative-related characteristics. We describe each class rather than assessing the association of a specific variable with class membership (i.e., there is not one independent variable of interest). As such, we do not report inferential statistics. Within the covariates used for descriptive statistics, the only missing data was on political party (*n*=3).

### Sensitivity analyses

Our primary analysis uses LCA input variables dichotomized on whether or not legislators rated a given characteristic as high priority (i.e., level 5) versus all other answers (levels 1–4). As a sensitivity analysis, we re-ran all of our analyses dichotomizing the inputs instead on the top two levels of priority (i.e., levels 4 and 5 versus levels 1–3).

### Software

We completed the majority of data processing and visualization in R version 4.1.2 and used SAS version 9.4 for the LCA (PROC LCA).

## Results

Full descriptive statistics for all included state legislators are shown in Table 1. Legislators were, on average, male (74.5%) and had spent an average of 9 years in the legislature. Legislators were 53% Republican and 46% Democrat (1.6% other), with 68% identifying as fiscally conservative, and 50% identifying as socially conservative. A majority (62.5%) reported sponsoring a health-related bill at some point. About one quarter were from the Northeast (23.5%), one quarter from the Midwest (25.4%), one third from the South (32.5%), 18% from the West, and 0.6% were from the territories or Puerto Rico. Most legislators had either a college degree (36%) or higher (45%).

**Table 1:** Descriptive statistics overall and stratified by latent class

	Constituent oriented decision makers (*N*=80)	Pragmatic consumers (*N*=308)	Uninterested skeptics (*N*=259)	Highly informed supporters (*N*=213)	Overall (*N*=862)
**Years in legislature**					
Mean (SD)	10.5 (9.19)	8.65 (7.05)	8.99 (8.20)	9.50 (8.13)	9.13 (7.89)
Median [Min, Max]	8.00 [0, 38.0]	6.00 [0, 40.0]	6.00 [0, 56.0]	7.00 [0, 40.0]	6.00 [0, 56.0]
**Gender**					
Female	23 (28.8%)	81 (26.3%)	69 (26.6%)	46 (21.6%)	220 (25.5%)
Male	57 (71.3%)	227 (73.7%)	190 (73.4%)	167 (78.4%)	642 (74.5%)
**Political party**					
Democrat	44 (55.0%)	135 (43.8%)	107 (41.3%)	104 (48.8%)	392 (45.5%)
Republican	36 (45.0%)	166 (53.9%)	148 (57.1%)	103 (48.4%)	453 (52.6%)
Other	0 (0%)	6 (1.9%)	2 (0.8%)	6 (2.8%)	14 (1.6%)
Missing	0 (0%)	1 (0.3%)	2 (0.8%)	0 (0%)	3 (0.3%)
**Reported ever sponsoring a health-related bill**					
Yes	48 (60.0%)	189 (61.4%)	159 (61.4%)	141 (66.2%)	539 (62.5%)
No	30 (37.5%)	112 (36.4%)	91 (35.1%)	68 (31.9%)	301 (34.9%)
Don’t know/Refused	2 (2.5%)	7 (2.3%)	9 (3.5%)	4 (1.9%)	22 (2.6%)
**Census region**					
Northeast	18 (22.5%)	72 (23.4%)	59 (22.8%)	53 (24.9%)	203 (23.5%)
Midwest	18 (22.5%)	86 (27.9%)	68 (26.3%)	47 (22.1%)	219 (25.4%)
South	26 (32.5%)	100 (32.5%)	78 (30.1%)	75 (35.2%)	280 (32.5%)
West	18 (22.5%)	50 (16.2%)	52 (20.1%)	35 (16.4%)	155 (18.0%)
Territories or Puerto Rico	0 (0%)	0 (0%)	2 (0.8%)	3 (1.4%)	5 (0.6%)
**Educational attainment**					
Less than college	21 (26.3%)	57 (18.5%)	59 (22.8%)	28 (13.1%)	165 (19.1%)
College	21 (26.3%)	111 (36.0%)	91 (35.1%)	86 (40.4%)	309 (35.8%)
More than college	38 (47.5%)	139 (45.1%)	107 (41.3%)	99 (46.5%)	385 (44.7%)
Don’t know/Refused	0 (0%)	1 (0.3%)	2 (0.8%)	0 (0%)	3 (0.3%)
**Self-rated stance on social issues**					
Liberal	27 (33.8%)	96 (31.2%)	52 (20.1%)	63 (29.6%)	239 (27.7%)
Moderate	14 (17.5%)	53 (17.2%)	59 (22.8%)	44 (20.7%)	171 (19.8%)
Conservative	37 (46.3%)	152 (49.4%)	141 (54.4%)	100 (46.9%)	430 (49.9%)
Other	0 (0%)	2 (0.6%)	1 (0.4%)	1 (0.5%)	4 (0.5%)
Don’t know/Refused	2 (2.5%)	5 (1.6%)	6 (2.3%)	5 (2.3%)	18 (2.1%)
**Self-rated stance on fiscal issues**					
Liberal	7 (8.8%)	31 (10.1%)	19 (7.3%)	32 (15.0%)	89 (10.3%)
Moderate	21 (26.3%)	62 (20.1%)	46 (17.8%)	46 (21.6%)	175 (20.3%)
Conservative	51 (63.8%)	212 (68.8%)	190 (73.4%)	130 (61.0%)	585 (67.9%)
Other	0 (0%)	0 (0%)	1 (0.4%)	1 (0.5%)	2 (0.2%)
Don’t know/Refused	1 (1.3%)	3 (1.0%)	3 (1.2%)	4 (1.9%)	11 (1.3%)
**Self-rated health**					
Excellent	15 (18.8%)	93 (30.2%)	70 (27.0%)	64 (30.0%)	243 (28.2%)
Not Excellent	65 (81.3%)	215 (69.8%)	186 (71.8%)	149 (70.0%)	616 (71.5%)
Don’t know/Refused	0 (0%)	0 (0%)	3 (1.2%)	0 (0%)	3 (0.3%)
**How often do you actively seek out research information when working on new policies?**					
Never	1 (1.3%)	0 (0%)	0 (0%)	0 (0%)	1 (0.1%)
Rarely	2 (2.5%)	3 (1.0%)	6 (2.3%)	4 (1.9%)	15 (1.7%)
Sometimes	17 (21.3%)	41 (13.3%)	64 (24.7%)	27 (12.7%)	149 (17.3%)
Most of the time	39 (48.8%)	155 (50.3%)	128 (49.4%)	102 (47.9%)	425 (49.3%)
Always	21 (26.3%)	109 (35.4%)	61 (23.6%)	79 (37.1%)	271 (31.4%)
Don’t know/refused	0 (0%)	0 (0%)	0 (0%)	1 (0.5%)	1 (0.1%)

**Fig. 1:**
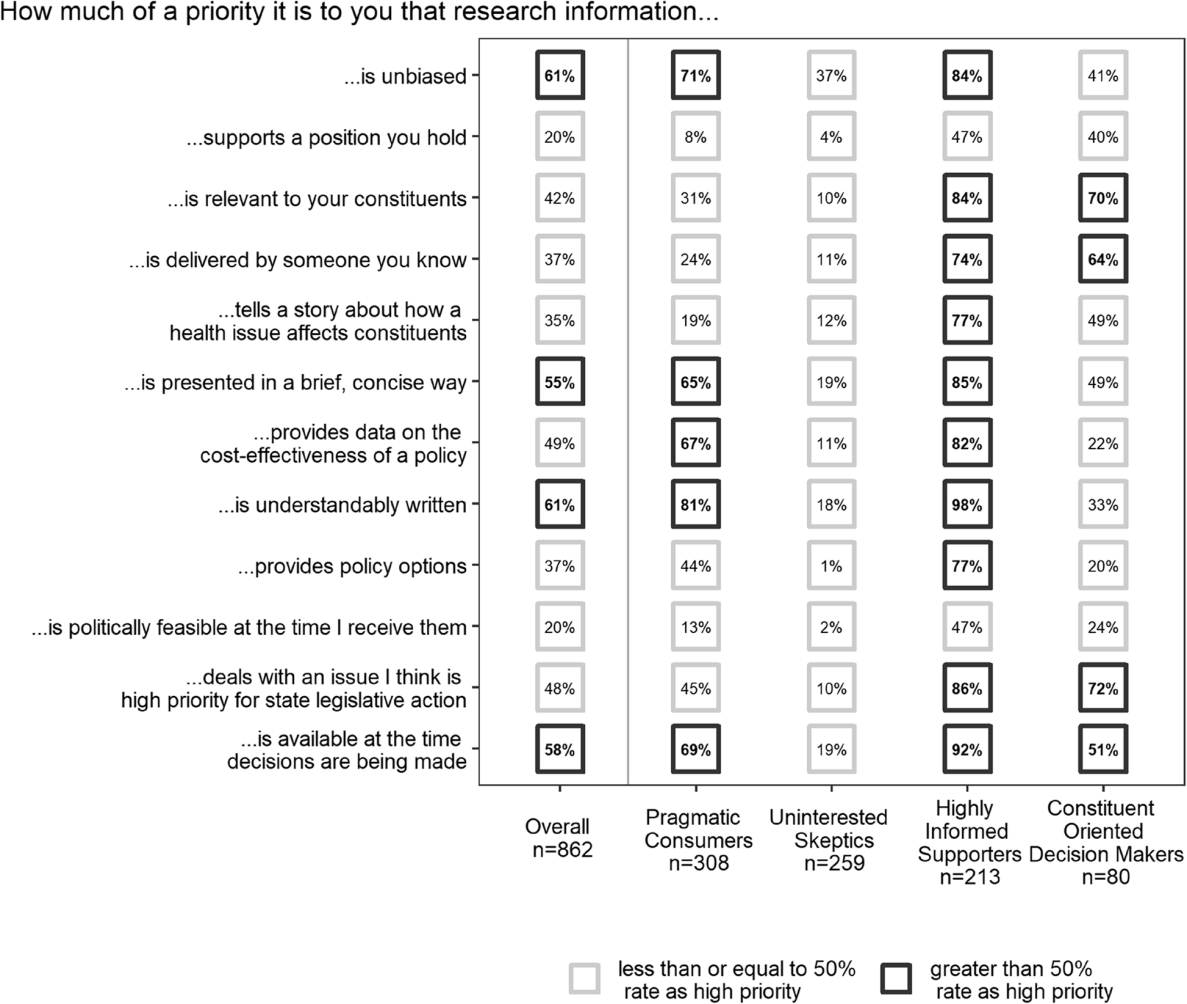
Percent of legislators rating a given research characteristic as high priority, displayed for the overall sample, and stratified by latent class. *Figure notes*: Number displayed within each point is the percentage of state legislators who reported that characteristic as high priority, conditional on the specific group. Percentages can theoretically range from zero to 100, and we present raw sample percentages for overall group, and latent class parameter estimates (i.e., conditional item-response probabilities) for class-stratified columns. Number of legislators in latent classes sums to 860 because 2 legislators were missing on all latent class analysis input variables

Overall descriptive statistics of the LCA input variables are displayed on the left side of Fig. 1. The majority of legislators placed a high priority on research that was brief and concise (55%), understandably written (61%), unbiased (61%), and available at the time that decisions are made (58%). The fewest legislators placed a high priority on research that supported a position they held (20%) or was politically feasible at the time it was received (20%).

The four-class solution was the best-fitting model based on model convergence, fit statistics, and interpretability. Models with 1–4 classes converged well (>80% of iterations converged on the same maximum likelihood solution), while only 67% of the 5 class iterations and 39% of 6 class solution iterations converged on the same solution. There was a clear decrease in information criteria statistics up until the four-class solution and change in fit statistics afterwards is small or even increasing (Additional file 1: Table S1 and Fig. S1). Finally, the four-class solution had clearly separable classes with distinct interpretations.

The right side of Fig. 1 displays the percentage of legislators within each class who rated a given characteristic as high priority, with values at least 50% bolded. We used these percentages to develop names for each class that captured their most highly prioritized characteristics of research.

The *Pragmatic Consumers* group was the most prevalent in our sample (*n*=308, 36%). This group prioritized research that was brief and concise (65%), provided cost-effectiveness information (67%), was understandably written (81%), unbiased (71%), and available at the time decisions were made (69%).

About 30% (*n*=259) of legislators belonged to the *Uninterested Skeptics* group. On average, legislators in this group did not place a high priority on any included research characteristics. The most highly prioritized characteristic was unbiasedness (37%).

One-quarter of legislators belonged to the *Highly Informed Supporters* group (*n*=213, 25%). The majority of this group placed on a high priority on all but two characteristics of research. The remaining two characteristics, research supporting a position they hold and being politically feasible, were prioritized by just under half of the sample (47% each).

Finally, the fewest legislators were classified as *Constituent-Oriented Decision Makers* (*n*=80, 9%). In general, this group prioritized characteristics of research related to their constituency or state: research that was relevant to their constituents (70%), delivered by someone they knew or trusted (64%), was available at the time decisions were made (51%), and dealt with an issue that they felt was a priority for state legislative action (72%).

Descriptive statistics stratified by class are shown in Table 1 and additional information on factors determining the health issues legislators work on, what sources are used when making policy decisions, and opinions about the reliability of research information are included in Additional file 1: Tables S2-S4.

We tested the sensitivity of these results by repeating all analyses with an alternative set of LCA input variables that dichotomized on whether a legislator rated a character as 4 or 5 (i.e., highest priority or second-from-highest priority) vs. less than 4. Practically, this meant that all of the LCA inputs were shifted in the “positive” direction (Table S5). The best fitting solution from this analysis was composed of three classes, rather than four as in the primary analysis (Table S1 and Fig. S1). First, there was one class with relatively low prioritization of all research characteristics, similar to the Uninterested Skeptics of our primary analyses; only research relevant to constituents, and delivered by someone they know or respect, was prioritized by at least 50% of legislators. Another group had extremely high prioritization of all research characteristics (most prioritized by over 90% of legislators), similar to the Highly Informed Supporters in our primary analyses. The third group also had a high prioritization of most characteristics except for research supporting a position they hold or being politically feasible when received. Comparing to our primary analysis, this group contains characteristics highly prioritized by the Pragmatic Consumers and Constituent-Oriented Decision Makers. The distribution of legislators into classes was also shifted towards more research-positive classes (Fig. S2). Full results including descriptive statistics are shown in Additional file 1: Fig. S2 and Tables S6-S9.

## Discussion

Our work indicates that state legislators have different preferences for sources and presentation of research information. We found four distinct groups of legislators. Broadly, these legislators either expressed some priorities and preferences for research or did not (i.e., the Uninterested Skeptics). Of those legislators who did express preferences for research, there were three general groups. Highly Informed Supporters prioritized nearly all of the characteristics we examined. More than one third of legislators (36%) in our work were classified as Pragmatic Consumers—legislators who prioritized research that was unbiased, available when needed, and included cost-effectiveness results. Some legislators were also classified as Constituent-Oriented Decision Makers. These legislators typically put a high priority on research that was relevant to their state and constituency, and it was important that research be delivered by someone they trusted.

Both skeptical and interested/enthusiastic groups of preferences have been found in previous work [16, 21]. A study using similar input variables, but different clustering methods, classified legislators as either enthusiastic or skeptical of research [21]. More recently, Purtle et al. used LCA and found a similar divide between skeptical and supportive legislators, specifically related to behavioral health research and legislation [16]. Taken together, there appears to be emerging consensus that some legislators will place a lower priority on research information, while others will prioritize research information more highly.

However, our work indicates that within those legislators who prioritize research characteristics there are different groups with nuanced preferences. Both Pragmatic Consumers and Constituent-Oriented Decision Makers average responses were essentially subsets of the Highly Informed Supporters’ preferences. This finding is also supported by findings in Purtle et al. [16]. Within supportive legislators—those who were supportive of behavioral health legislation and less skeptical about treatment options—there were both “action-oriented” and “passive” supporters [16]. One of the starkest differences between those groups of legislators was that at least 90% of “action-oriented” supports had introduced a behavioral health bill, compared to at most 23% of “passive” supporters.

### Implications for research dissemination

This analysis drives home the fact that communicating research cannot be a one-size-fits-all approach. For dissemination efforts to be successful, researchers must consider how to communicate with legislators who have different preferences, levels of understanding, values, and priorities. For example, policy briefs are a widely used dissemination tool, and researchers and advocates should take care to purposefully construct policy briefs in a way designed to appeal to a broad range of legislators, who our work shows have distinct priorities for research information.

In the immediate future, public health practitioners and advocates can work to ensure that policy briefs are designed to (a) have broad appeal and (b) incorporate specific aspects to engage policymakers with different preferences. To have broad appeal, our work reinforces the need for concise and understandable briefs written using unbiased language. Also, in line with mainstream policy dissemination theories, information will be most useful to decision makers during the “window of opportunity”—i.e.., when a related health issue is of major interest and political will for change exists [23].

Beyond these broad characteristics, briefs should also incorporate pieces designed to engage policymakers who have different preferences. This suggestion could be operationalized in multiple ways. For example, including a section on potential costs and cost-effectiveness might appeal most to Pragmatic Consumers. Opening a brief by clearly outlining how a health issue affects constituents could be used to engage Constituent-Oriented Decision Makers. Highly Informed Supporters may most appreciate a story on how the health issue affects constituents or information on multiple policy options to address a health issue. Future research could empirically examine which themes might resonate with different groups of legislators in the context of a policy brief.

Practitioners and advocates should expect uninterested legislators based on our findings and previous research; alternative communication or engagement strategies will likely be needed for these legislators. Nearly one third of our analytic sample was classified as Uninterested Skeptics—researchers who generally did not place a high priority on any research presentation characteristics. Engaging policymakers in the research process is generally known to be an important facilitator of research use [9–12], and this might be a particularly important way to engage skeptical decision makers, build trust, and facilitate the use of research information once produced. Indeed, our considerations for presenting research information should be coupled with other dissemination strategies, such as engaging policymakers directly, knowing the policy players, using knowledge brokers, and drumming up support from experts and advocates [12].

Finally, advocates might be interested in purposefully disseminating different briefs to legislators based on how we expect they would prefer to receive information. This type of approach is supported by previous work that has indicated that specific briefs are more likely to be useful for specific policymakers [13]. For example, making the research’s links to a specific constituency very clear and having a well-known researcher or advocate deliver the brief might improve its potential to Constituent-Oriented Decision Makers. Presenting a brief entirely on cost-effectiveness findings might be most valued by Pragmatic Consumers. However, this approach is more difficult without clear information relating expectations for research information to observable demographic or political characteristics, and more work is needed in that space. Linking these classes to individuals also related to a major interpretation concern in latent class analysis—that of reification (when latent class response profiles are assumed to represent individuals, rather than descriptions of averages) [24]. These classes are not intended to represent individuals; rather, they are a way to understand similar profiles of responses and better use that heterogeneity in our communications.

### Strengths and limitations

Our work is an important step forward in researchers’ and advocates’ ability to tailor communication materials and to structure our thinking around purposeful, audience-informed dissemination techniques. We used a dataset that offered rich information on researchers’ stated preferences as well as contextual information to characterize groups, and our findings are applicable across content areas. Additionally, LCA methods are an improvement over examining individual variables because they allow researchers to consider a wide range of inputs and take the relationships between those inputs into account. An important caveat to this and most other survey work with legislators is our focus on stated preferences that were assessed via telephone survey (i.e., what legislators say they prioritize), rather than revealed preferences (i.e., how legislators act). If legislators’ actions are aligned with their stated preferences, then these results should hold.

The data used here includes 46% of the randomly selected legislators. This response rate is in line with (or better than) other research sampling legislators [16, 25, 26], but sample selection may still be of concern. However, our analyses are still useful because they provide a comprehensive look at what legislators say they prefer, and the variation in those preferences, though the results may only be applicable to legislators like the 46% who responded. Given the main purpose of this research is to inform how broad messages can include specific components that are interesting to different groups of legislators, we do not see this selection as a major issue. Finally, while the data is less than 10 years old, the political environment of the USA has grown increasingly polarized [27]. It is possible that results have shifted; for example, similar groups may still exist but in different relative proportions. Thus, it will be critical to examine whether these groups reproduce in contemporary datasets. This is particularly true in an age when policy disinformation is rampant in the USA [28].

### Future directions

A key question for future research is how to engage Uninterested Skeptics. For example, if these folks do not prioritize the variables examined here, what do they prioritize? Are these individuals proposing health-related policies, and if so, in what areas? How did they decide to focus on those areas? It will also be important to rigorously examine how best to operationalize the research characteristics we examined. While it is clear that brief, unbiased, and well-written communications matter to decision makers, what convinces them that work is unbiased? What length of communication do they consider brief? What is the relative importance of the message versus the messenger? Another critical methodological question to move the field of dissemination research forward is to examine the relationship between legislators’ stated and revealed preferences—for example, do legislators who report valuing research incorporate it into their legislative agenda more often?

Finally, our input variables focused on priorities for presentation, without focusing on the delivery medium for that presentation (e.g., paper, in-person, email). Previous work has indicated that delivery medium preferences are likely different for different policymakers (e.g., legislator, staffer, executive) [13, 29]. As communications are increasingly virtual and mobile phones are critical communication tools, we need to better understand what types of communication mediums legislators prefer.

## Conclusions

Our findings add to the growing consensus that researchers and advocates should purposefully frame materials and messages (e.g., policy briefs, press releases) to include aspects that may catch the interest of policymakers with varied preferences. To that end, our data can be used to inform the specific content included in communication materials. More work is needed to inform how to better engage legislators who are uninterested in research and investigate the utility of stated preference methods.

## Supplementary Information

**Additional file 1: Table S1.** LCA fit statistics. **Figure S1.** LCA information criteria. **Table S2.** Two of seven factors that most help to determine which health issues you work on, primary analysis. **Table S3.** Use of specific sources when making policy decisions, primary analysis. **Table S4.** Reliability of research information, primary analysis. **Table S5.** Comparison with primary analytic inputs. **Figure S2.** LCA results, sensitivity analysis. **Table S6.** Demographic and political variables﻿, sensitivity analysis. **Table S7.** Two of seven factors that most help to determine which health issues you work on﻿, sensitivity analysis. **Table S8.** Use of specific sources when making policy decisions﻿, sensitivity analysis. **Table S9.** Reliability of research information﻿, sensitivity analysis.

## Data Availability

The data are available from the authors upon reasonable request.
