# Supplementary material for: Opportunities to improve policy dissemination by tailoring communication materials to the research priorities of legislators
Source: Implement Sci Commun. 2022 Mar 4;3:24. doi: 10.1186/s43058-022-00274-6 (PMC8895761; doi:10.1186/s43058-022-00274-6)
Supplement: Supplementary file 1 — Additional file 1: Table S1. LCA fit statistics. Figure S1. LCA information criteria. Table S2. Two of seven factors that most help to determine which health issues you work on, primary analysis. Table S3. Use of specific sources when making policy decisions, primary analysis. Table S4. Reliability of research information, primary analysis. Table S5. Comparison with primary analytic inputs. Figure S2. LCA results, sensitivity analysis. Table S6. Demographic and political variables﻿, sensitivity analysis. Table S7. Two of seven factors that most help to determine which health issues you work on﻿, sensitivity analysis. Table S8. Use of specific sources when making policy decisions﻿, sensitivity analysis. Table S9. Reliability of research information﻿, sensitivity analysis. [file 43058_2022_274_MOESM1_ESM.docx]

Appendix material for: “Opportunities to improve policy dissemination by tailoring communication materials to the research priorities of legislators”

Authors: Natalie R Smith ([nataliesmith@hsph.harvard.edu](mailto:nataliesmith@hsph.harvard.edu)), Stephanie Mazzucca, Marissa G Hall, Kristen Hassmiller Lich, Ross C Brownson, and Leah Frerichs

Table of Contents:

[Model Selection 2](#_Toc93141915)

[Table S1: LCA fit statistics 2](#_Toc93141916)

[Figure S1: LCA information criteria 2](#_Toc93141917)

[Primary analyses, supplementary descriptive statistics 3](#_Toc93141918)

[Table S2: Two of seven factors that most help to determine which health issues you work on 3](#_Toc93141919)

[Table S3: Use of specific sources when making policy decisions 4](#_Toc93141920)

[Table S4: Reliability of research information 6](#_Toc93141921)

[Sensitivity analyses 8](#_Toc93141922)

[Table S5: Comparison with primary analytic inputs 8](#_Toc93141923)

[Figure S2: LCA results 8](#_Toc93141924)

[Descriptive Statistics 9](#_Toc93141925)

[Table S6: Demographic and political variables 9](#_Toc93141926)

[Table S7: Two of seven factors that most help to determine which health issues you work on 11](#_Toc93141927)

[Table S8: Use of specific sources when making policy decisions 12](#_Toc93141928)

[Table S9: Reliability of research information 14](#_Toc93141929)

[Survey Instrument 16](#_Toc93141930)

# Model Selection

## Table S1: LCA fit statistics

| dataset | numClasses | AIC | BIC | pctConverging |
| --- | --- | --- | --- | --- |
| Dichotomize on 5 | 1 | 3,382.213 | 3,439.296 | 100.0 |
|  | 2 | 1,995.711 | 2,114.634 | 100.0 |
|  | 3 | 1,811.109 | 1,991.873 | 87.6 |
|  | 4 | 1,728.035 | 1,970.638 | 99.2 |
|  | 5 | 1,711.438 | 2,015.881 | 67.0 |
|  | 6 | 1,695.753 | 2,062.036 | 38.7 |
| Dichtomize on 45 | 1 | 2,191.582 | 2,248.665 | 100.0 |
|  | 2 | 1,467.088 | 1,586.012 | 100.0 |
|  | 3 | 1,366.504 | 1,547.267 | 99.9 |
|  | 4 | 1,331.870 | 1,574.473 | 98.1 |
|  | 5 | 1,322.442 | 1,626.886 | 1.3 |
|  | 6 | 1,317.863 | 1,684.147 | 1.4 |

## Figure S1: LCA information criteria


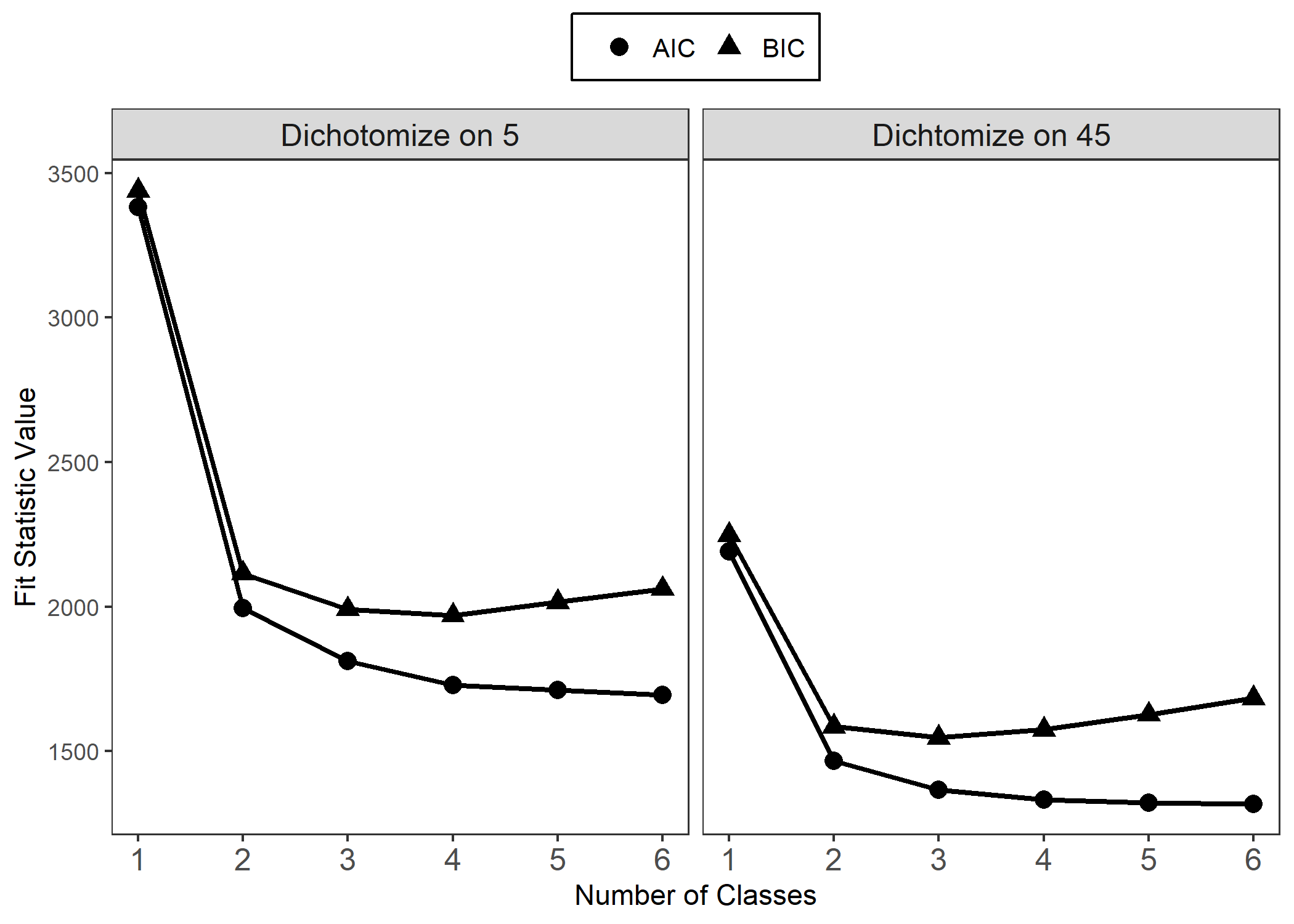


# Primary analyses, supplementary descriptive statistics

## Table S2: Two of seven factors that most help to determine which health issues you work on

Specific question wording: “I’d like to know how important the following factors are in determining what health issues you work on. I will read you a list and ask that you tell me the top two factors.”

| Two factors that most help determine which health issues you work on | Constituent   Oriented   Decision Makers (N=80) | Pragmatic   Consumers (N=308) | Uninterested   Skeptics (N=259) | Highly   Informed   Supporters (N=213) | Overall (N=862) |
| --- | --- | --- | --- | --- | --- |
| **Legislation being proposed by your colleagues** | | | | | |
| Yes | 21 (26.3%) | 108 (35.1%) | 87 (33.6%) | 48 (22.5%) | 264 (30.6%) |
| No | 57 (71.3%) | 191 (62.0%) | 169 (65.3%) | 161 (75.6%) | 580 (67.3%) |
| Don't know/Refused | 2 (2.5%) | 9 (2.9%) | 3 (1.2%) | 4 (1.9%) | 18 (2.1%) |
| **Personal interest** | | | | | |
| Yes | 22 (27.5%) | 66 (21.4%) | 47 (18.1%) | 54 (25.4%) | 189 (21.9%) |
| No | 56 (70.0%) | 233 (75.6%) | 209 (80.7%) | 155 (72.8%) | 655 (76.0%) |
| Don't know/Refused | 2 (2.5%) | 9 (2.9%) | 3 (1.2%) | 4 (1.9%) | 18 (2.1%) |
| **Research information** | | | | | |
| Yes | 12 (15.0%) | 70 (22.7%) | 53 (20.5%) | 50 (23.5%) | 185 (21.5%) |
| No | 66 (82.5%) | 229 (74.4%) | 203 (78.4%) | 159 (74.6%) | 659 (76.5%) |
| Don't know/Refused | 2 (2.5%) | 9 (2.9%) | 3 (1.2%) | 4 (1.9%) | 18 (2.1%) |
| **Constituents' needs and opinions** | | | | | |
| Yes | 54 (67.5%) | 195 (63.3%) | 175 (67.6%) | 145 (68.1%) | 571 (66.2%) |
| No | 24 (30.0%) | 104 (33.8%) | 81 (31.3%) | 64 (30.0%) | 273 (31.7%) |
| Don't know/Refused | 2 (2.5%) | 9 (2.9%) | 3 (1.2%) | 4 (1.9%) | 18 (2.1%) |
| **Data impacting your local area** | | | | | |
| Yes | 22 (27.5%) | 82 (26.6%) | 78 (30.1%) | 63 (29.6%) | 245 (28.4%) |
| No | 56 (70.0%) | 217 (70.5%) | 178 (68.7%) | 146 (68.5%) | 599 (69.5%) |
| Don't know/Refused | 2 (2.5%) | 9 (2.9%) | 3 (1.2%) | 4 (1.9%) | 18 (2.1%) |
| **Interaction with lobbyists** | | | | | |
| Yes | 8 (10.0%) | 19 (6.2%) | 22 (8.5%) | 14 (6.6%) | 64 (7.4%) |
| No | 70 (87.5%) | 280 (90.9%) | 234 (90.3%) | 195 (91.5%) | 780 (90.5%) |
| Don't know/Refused | 2 (2.5%) | 9 (2.9%) | 3 (1.2%) | 4 (1.9%) | 18 (2.1%) |
| **Economic issues** |  |  |  |  |  |
| Yes | 16 (20.0%) | 50 (16.2%) | 46 (17.8%) | 41 (19.2%) | 154 (17.9%) |
| No | 62 (77.5%) | 249 (80.8%) | 210 (81.1%) | 168 (78.9%) | 690 (80.0%) |
| Don't know/Refused | | | | | |

## Table S3: Use of specific sources when making policy decisions

Specific question wording: “We know that policymakers must take a number of factors into account when making policy decisions. Now I’d like to ask you a few questions about how you use research information when you are working on an issue. I will read a series of statements and ask that you tell me, on a scale of 1 to 5, how often you use these things when you are making policy decisions. One means you never use them and 5 means you always use them. How often do you:”

| When making policy decisions, how often do you... | Constituent   Oriented   Decision Makers (N=80) | Pragmatic   Consumers (N=308) | Uninterested   Skeptics (N=259) | Highly   Informed   Supporters (N=213) | Overall (N=862) |
| --- | --- | --- | --- | --- | --- |
| **...explore what other states are doing on the issue** | | | | | |
| Never | 0 (0%) | 6 (1.9%) | 8 (3.1%) | 4 (1.9%) | 18 (2.1%) |
| 2 | 7 (8.8%) | 30 (9.7%) | 30 (11.6%) | 12 (5.6%) | 79 (9.2%) |
| 3 | 30 (37.5%) | 92 (29.9%) | 99 (38.2%) | 66 (31.0%) | 287 (33.3%) |
| 4 | 27 (33.8%) | 111 (36.0%) | 96 (37.1%) | 65 (30.5%) | 299 (34.7%) |
| Always | 16 (20.0%) | 69 (22.4%) | 26 (10.0%) | 65 (30.5%) | 178 (20.6%) |
| Not sure/Refused | 0 (0%) | 0 (0%) | 0 (0%) | 1 (0.5%) | 1 (0.1%) |
| **...read scientific research reports on the issue** | | | | | |
| Never | 4 (5.0%) | 21 (6.8%) | 19 (7.3%) | 17 (8.0%) | 61 (7.1%) |
| 2 | 14 (17.5%) | 48 (15.6%) | 67 (25.9%) | 38 (17.8%) | 167 (19.4%) |
| 3 | 20 (25.0%) | 79 (25.6%) | 82 (31.7%) | 46 (21.6%) | 227 (26.3%) |
| 4 | 23 (28.8%) | 105 (34.1%) | 73 (28.2%) | 65 (30.5%) | 266 (30.9%) |
| Always | 18 (22.5%) | 53 (17.2%) | 18 (6.9%) | 47 (22.1%) | 138 (16.0%) |
| Not sure/Refused | 1 (1.3%) | 2 (0.6%) | 0 (0%) | 0 (0%) | 3 (0.3%) |
| **...read or watch popular media stories on the issue** | | | | | |
| Never | 9 (11.3%) | 57 (18.5%) | 40 (15.4%) | 26 (12.2%) | 132 (15.3%) |
| 2 | 18 (22.5%) | 89 (28.9%) | 76 (29.3%) | 43 (20.2%) | 228 (26.5%) |
| 3 | 25 (31.3%) | 85 (27.6%) | 79 (30.5%) | 60 (28.2%) | 249 (28.9%) |
| 4 | 16 (20.0%) | 58 (18.8%) | 50 (19.3%) | 50 (23.5%) | 174 (20.2%) |
| Always | 12 (15.0%) | 17 (5.5%) | 11 (4.2%) | 34 (16.0%) | 74 (8.6%) |
| Not sure/Refused | 0 (0%) | 2 (0.6%) | 3 (1.2%) | 0 (0%) | 5 (0.6%) |
| **...ask internal legislative research bureaus for information on the issue** | | | | | |
| Never | 2 (2.5%) | 6 (1.9%) | 9 (3.5%) | 6 (2.8%) | 23 (2.7%) |
| 2 | 6 (7.5%) | 14 (4.5%) | 18 (6.9%) | 10 (4.7%) | 48 (5.6%) |
| 3 | 15 (18.8%) | 44 (14.3%) | 58 (22.4%) | 19 (8.9%) | 136 (15.8%) |
| 4 | 32 (40.0%) | 106 (34.4%) | 100 (38.6%) | 44 (20.7%) | 283 (32.8%) |
| Always | 25 (31.3%) | 136 (44.2%) | 72 (27.8%) | 133 (62.4%) | 367 (42.6%) |
| Not sure/Refused | 0 (0%) | 2 (0.6%) | 2 (0.8%) | 1 (0.5%) | 5 (0.6%) |
| **...ask external legislative research organization for information on the issue** | | | | | |
| Never | 7 (8.8%) | 17 (5.5%) | 23 (8.9%) | 14 (6.6%) | 62 (7.2%) |
| 2 | 12 (15.0%) | 44 (14.3%) | 61 (23.6%) | 20 (9.4%) | 138 (16.0%) |
| 3 | 27 (33.8%) | 101 (32.8%) | 81 (31.3%) | 40 (18.8%) | 249 (28.9%) |
| 4 | 27 (33.8%) | 99 (32.1%) | 73 (28.2%) | 72 (33.8%) | 271 (31.4%) |
| Always | 7 (8.8%) | 45 (14.6%) | 20 (7.7%) | 66 (31.0%) | 138 (16.0%) |
| Not sure/Refused | 0 (0%) | 2 (0.6%) | 1 (0.4%) | 1 (0.5%) | 4 (0.5%) |
| **...attend seminars or presentations where research is discussed** | | | | | |
| Never | 9 (11.3%) | 42 (13.6%) | 41 (15.8%) | 18 (8.5%) | 110 (12.8%) |
| 2 | 22 (27.5%) | 90 (29.2%) | 72 (27.8%) | 52 (24.4%) | 236 (27.4%) |
| 3 | 25 (31.3%) | 96 (31.2%) | 98 (37.8%) | 57 (26.8%) | 278 (32.3%) |
| 4 | 18 (22.5%) | 63 (20.5%) | 39 (15.1%) | 60 (28.2%) | 180 (20.9%) |
| Always | 6 (7.5%) | 16 (5.2%) | 8 (3.1%) | 26 (12.2%) | 56 (6.5%) |
| Not sure/Refused | 0 (0%) | 1 (0.3%) | 1 (0.4%) | 0 (0%) | 2 (0.2%) |
| **...contact scientific researchers or experts for advice** | | | | | |
| Never | 11 (13.8%) | 45 (14.6%) | 39 (15.1%) | 20 (9.4%) | 115 (13.3%) |
| 2 | 23 (28.8%) | 86 (27.9%) | 60 (23.2%) | 45 (21.1%) | 215 (24.9%) |
| 3 | 17 (21.3%) | 84 (27.3%) | 102 (39.4%) | 49 (23.0%) | 252 (29.2%) |
| 4 | 21 (26.3%) | 68 (22.1%) | 44 (17.0%) | 56 (26.3%) | 189 (21.9%) |
| Always | 8 (10.0%) | 22 (7.1%) | 13 (5.0%) | 41 (19.2%) | 84 (9.7%) |
| Not sure/Refused | 0 (0%) | 3 (1.0%) | 1 (0.4%) | 2 (0.9%) | 7 (0.8%) |
| **...take the results of a relevant scientific study into account when making a decision** | | | | | |
| Never | 0 (0%) | 6 (1.9%) | 7 (2.7%) | 3 (1.4%) | 16 (1.9%) |
| 2 | 6 (7.5%) | 12 (3.9%) | 22 (8.5%) | 6 (2.8%) | 46 (5.3%) |
| 3 | 11 (13.8%) | 53 (17.2%) | 65 (25.1%) | 23 (10.8%) | 152 (17.6%) |
| 4 | 41 (51.3%) | 142 (46.1%) | 121 (46.7%) | 76 (35.7%) | 380 (44.1%) |
| Always | 22 (27.5%) | 92 (29.9%) | 41 (15.8%) | 103 (48.4%) | 260 (30.2%) |
| Not sure/Refused | 0 (0%) | 3 (1.0%) | 3 (1.2%) | 2 (0.9%) | 8 (0.9%) |
| **...talk with your colleagues about research on issues important to you** | | | | | |
| Never | 1 (1.3%) | 3 (1.0%) | 2 (0.8%) | 2 (0.9%) | 8 (0.9%) |
| 2 | 3 (3.8%) | 12 (3.9%) | 22 (8.5%) | 7 (3.3%) | 44 (5.1%) |
| 3 | 16 (20.0%) | 52 (16.9%) | 57 (22.0%) | 22 (10.3%) | 148 (17.2%) |
| 4 | 16 (20.0%) | 109 (35.4%) | 116 (44.8%) | 44 (20.7%) | 286 (33.2%) |
| Always | 44 (55.0%) | 130 (42.2%) | 62 (23.9%) | 138 (64.8%) | 374 (43.4%) |
| Not sure/Refused | 0 (0%) | 2 (0.6%) | 0 (0%) | 0 (0%) | 2 (0.2%) |
| **...use research to justify a decision you made** | | | | | |
| Never | 1 (1.3%) | 10 (3.2%) | 5 (1.9%) | 2 (0.9%) | 18 (2.1%) |
| 2 | 1 (1.3%) | 12 (3.9%) | 15 (5.8%) | 1 (0.5%) | 29 (3.4%) |
| 3 | 15 (18.8%) | 32 (10.4%) | 56 (21.6%) | 18 (8.5%) | 121 (14.0%) |
| 4 | 30 (37.5%) | 133 (43.2%) | 136 (52.5%) | 59 (27.7%) | 358 (41.5%) |
| Always | 31 (38.8%) | 117 (38.0%) | 47 (18.1%) | 131 (61.5%) | 328 (38.1%) |
| Not sure/Refused | 2 (2.5%) | 4 (1.3%) | 0 (0%) | 2 (0.9%) | 8 (0.9%) |
| **...use research presented in committee testimony** | | | | | |
| Never | 0 (0%) | 9 (2.9%) | 1 (0.4%) | 1 (0.5%) | 11 (1.3%) |
| 2 | 1 (1.3%) | 14 (4.5%) | 21 (8.1%) | 4 (1.9%) | 41 (4.8%) |
| 3 | 11 (13.8%) | 50 (16.2%) | 53 (20.5%) | 26 (12.2%) | 140 (16.2%) |
| 4 | 37 (46.3%) | 105 (34.1%) | 121 (46.7%) | 54 (25.4%) | 317 (36.8%) |
| Always | 31 (38.8%) | 127 (41.2%) | 60 (23.2%) | 127 (59.6%) | 346 (40.1%) |
| Not sure/Refused | 0 (0%) | 3 (1.0%) | 3 (1.2%) | 1 (0.5%) | 7 (0.8%) |

## Table S4: Reliability of research information

Specific question wording: “Now I would like to know how reliable you believe research information is when it comes from different sources. On a scale of 1 to 5 with 1 meaning very unreliable and 5 meaning very reliable, please tell me how important it is to you that research information comes from:”

| How reliable research information is to you when it comes from ... | Constituent   Oriented   Decision Makers (N=80) | Pragmatic   Consumers (N=308) | Uninterested   Skeptics (N=259) | Highly   Informed   Supporters (N=213) | Overall (N=862) |
| --- | --- | --- | --- | --- | --- |
| **... A university** |  |  |  |  |  |
| Very unreliable | 0 (0%) | 3 (1.0%) | 2 (0.8%) | 2 (0.9%) | 7 (0.8%) |
| 2 | 7 (8.8%) | 10 (3.2%) | 16 (6.2%) | 4 (1.9%) | 37 (4.3%) |
| 3 | 14 (17.5%) | 86 (27.9%) | 77 (29.7%) | 46 (21.6%) | 223 (25.9%) |
| 4 | 39 (48.8%) | 141 (45.8%) | 125 (48.3%) | 64 (30.0%) | 371 (43.0%) |
| Very reliable | 20 (25.0%) | 64 (20.8%) | 36 (13.9%) | 92 (43.2%) | 212 (24.6%) |
| Not sure/Refused | 0 (0%) | 4 (1.3%) | 3 (1.2%) | 5 (2.3%) | 12 (1.4%) |
| **... A government source** |  |  |  |  |  |
| Very unreliable | 2 (2.5%) | 9 (2.9%) | 14 (5.4%) | 11 (5.2%) | 36 (4.2%) |
| 2 | 6 (7.5%) | 39 (12.7%) | 47 (18.1%) | 16 (7.5%) | 109 (12.6%) |
| 3 | 32 (40.0%) | 129 (41.9%) | 116 (44.8%) | 83 (39.0%) | 360 (41.8%) |
| 4 | 36 (45.0%) | 110 (35.7%) | 69 (26.6%) | 71 (33.3%) | 287 (33.3%) |
| Very reliable | 3 (3.8%) | 16 (5.2%) | 9 (3.5%) | 26 (12.2%) | 54 (6.3%) |
| Not sure/Refused | 1 (1.3%) | 5 (1.6%) | 4 (1.5%) | 6 (2.8%) | 16 (1.9%) |
| **... Industry** |  |  |  |  |  |
| Very unreliable | 4 (5.0%) | 11 (3.6%) | 7 (2.7%) | 5 (2.3%) | 28 (3.2%) |
| 2 | 13 (16.3%) | 55 (17.9%) | 57 (22.0%) | 34 (16.0%) | 159 (18.4%) |
| 3 | 34 (42.5%) | 143 (46.4%) | 114 (44.0%) | 93 (43.7%) | 385 (44.7%) |
| 4 | 25 (31.3%) | 85 (27.6%) | 76 (29.3%) | 51 (23.9%) | 237 (27.5%) |
| Very reliable | 4 (5.0%) | 10 (3.2%) | 4 (1.5%) | 27 (12.7%) | 45 (5.2%) |
| Not sure/Refused | 0 (0%) | 4 (1.3%) | 1 (0.4%) | 3 (1.4%) | 8 (0.9%) |
| **... Advocacy groups** |  |  |  |  |  |
| Very unreliable | 4 (5.0%) | 26 (8.4%) | 24 (9.3%) | 13 (6.1%) | 67 (7.8%) |
| 2 | 18 (22.5%) | 76 (24.7%) | 80 (30.9%) | 32 (15.0%) | 208 (24.1%) |
| 3 | 42 (52.5%) | 149 (48.4%) | 113 (43.6%) | 104 (48.8%) | 408 (47.3%) |
| 4 | 10 (12.5%) | 47 (15.3%) | 37 (14.3%) | 53 (24.9%) | 147 (17.1%) |
| Very reliable | 6 (7.5%) | 2 (0.6%) | 1 (0.4%) | 7 (3.3%) | 16 (1.9%) |
| Not sure/Refused | 0 (0%) | 8 (2.6%) | 4 (1.5%) | 4 (1.9%) | 16 (1.9%) |
| **... Constituents** |  |  |  |  |  |
| Very unreliable | 0 (0%) | 5 (1.6%) | 8 (3.1%) | 6 (2.8%) | 20 (2.3%) |
| 2 | 5 (6.3%) | 38 (12.3%) | 31 (12.0%) | 13 (6.1%) | 87 (10.1%) |
| 3 | 29 (36.3%) | 119 (38.6%) | 109 (42.1%) | 81 (38.0%) | 338 (39.2%) |
| 4 | 25 (31.3%) | 111 (36.0%) | 90 (34.7%) | 62 (29.1%) | 288 (33.4%) |
| Very reliable | 21 (26.3%) | 30 (9.7%) | 18 (6.9%) | 46 (21.6%) | 115 (13.3%) |
| Not sure/Refused | 0 (0%) | 5 (1.6%) | 3 (1.2%) | 5 (2.3%) | 14 (1.6%) |
| **... The media** |  |  |  |  |  |
| Very unreliable | 14 (17.5%) | 79 (25.6%) | 64 (24.7%) | 53 (24.9%) | 212 (24.6%) |
| 2 | 26 (32.5%) | 121 (39.3%) | 112 (43.2%) | 72 (33.8%) | 331 (38.4%) |
| 3 | 29 (36.3%) | 93 (30.2%) | 74 (28.6%) | 74 (34.7%) | 270 (31.3%) |
| 4 | 11 (13.8%) | 12 (3.9%) | 6 (2.3%) | 10 (4.7%) | 39 (4.5%) |
| Very reliable | 0 (0%) | 0 (0%) | 0 (0%) | 1 (0.5%) | 1 (0.1%) |
| Not sure/Refused | 0 (0%) | 3 (1.0%) | 3 (1.2%) | 3 (1.4%) | 9 (1.0%) |
| **... Other legislators** |  |  |  |  |  |
| Very unreliable | 3 (3.8%) | 3 (1.0%) | 1 (0.4%) | 5 (2.3%) | 12 (1.4%) |
| 2 | 4 (5.0%) | 37 (12.0%) | 27 (10.4%) | 10 (4.7%) | 79 (9.2%) |
| 3 | 27 (33.8%) | 144 (46.8%) | 136 (52.5%) | 90 (42.3%) | 397 (46.1%) |
| 4 | 40 (50.0%) | 107 (34.7%) | 89 (34.4%) | 75 (35.2%) | 312 (36.2%) |
| Very reliable | 4 (5.0%) | 13 (4.2%) | 4 (1.5%) | 27 (12.7%) | 48 (5.6%) |
| Not sure/Refused | 2 (2.5%) | 4 (1.3%) | 2 (0.8%) | 6 (2.8%) | 14 (1.6%) |
| **... Caucus leadership** |  |  |  |  |  |
| Very unreliable | 4 (5.0%) | 25 (8.1%) | 16 (6.2%) | 15 (7.0%) | 60 (7.0%) |
| 2 | 6 (7.5%) | 47 (15.3%) | 48 (18.5%) | 21 (9.9%) | 123 (14.3%) |
| 3 | 25 (31.3%) | 122 (39.6%) | 102 (39.4%) | 77 (36.2%) | 326 (37.8%) |
| 4 | 32 (40.0%) | 93 (30.2%) | 82 (31.7%) | 71 (33.3%) | 279 (32.4%) |
| Very reliable | 11 (13.8%) | 11 (3.6%) | 8 (3.1%) | 23 (10.8%) | 53 (6.1%) |
| Not sure/Refused | 2 (2.5%) | 10 (3.2%) | 3 (1.2%) | 6 (2.8%) | 21 (2.4%) |

# Sensitivity analyses

## Table S5: Comparison with primary analytic inputs

| LCA input | pct5other | pct45other | difference |
| --- | --- | --- | --- |
| ...is unbiased | 61.2 | 79.6 | 18.4 |
| ...supports a position you hold | 20.1 | 47.2 | 27.1 |
| ...is relevant to your constituents | 42.5 | 82.1 | 39.6 |
| ...is delivered by someone you know | 37.5 | 79.2 | 41.7 |
| ...tells a story about how a   health issue affects constituents | 34.8 | 75.2 | 40.4 |
| ...is presented in a brief, concise way | 54.7 | 84.1 | 29.4 |
| ...provides data on the   cost-effectiveness of a policy | 49.4 | 82.0 | 32.6 |
| ...is understandably written | 61.1 | 87.0 | 25.9 |
| ...provides policy options | 37.3 | 75.9 | 38.6 |
| ...is politically feasible at the time I receive them | 19.6 | 49.3 | 29.7 |
| ...deals with an issue I think is   high priority for state legislative action | 48.3 | 84.3 | 36.0 |
| ...is available at the time   decisions are being made | 58.2 | 86.6 | 28.4 |

## Figure S2: LCA results


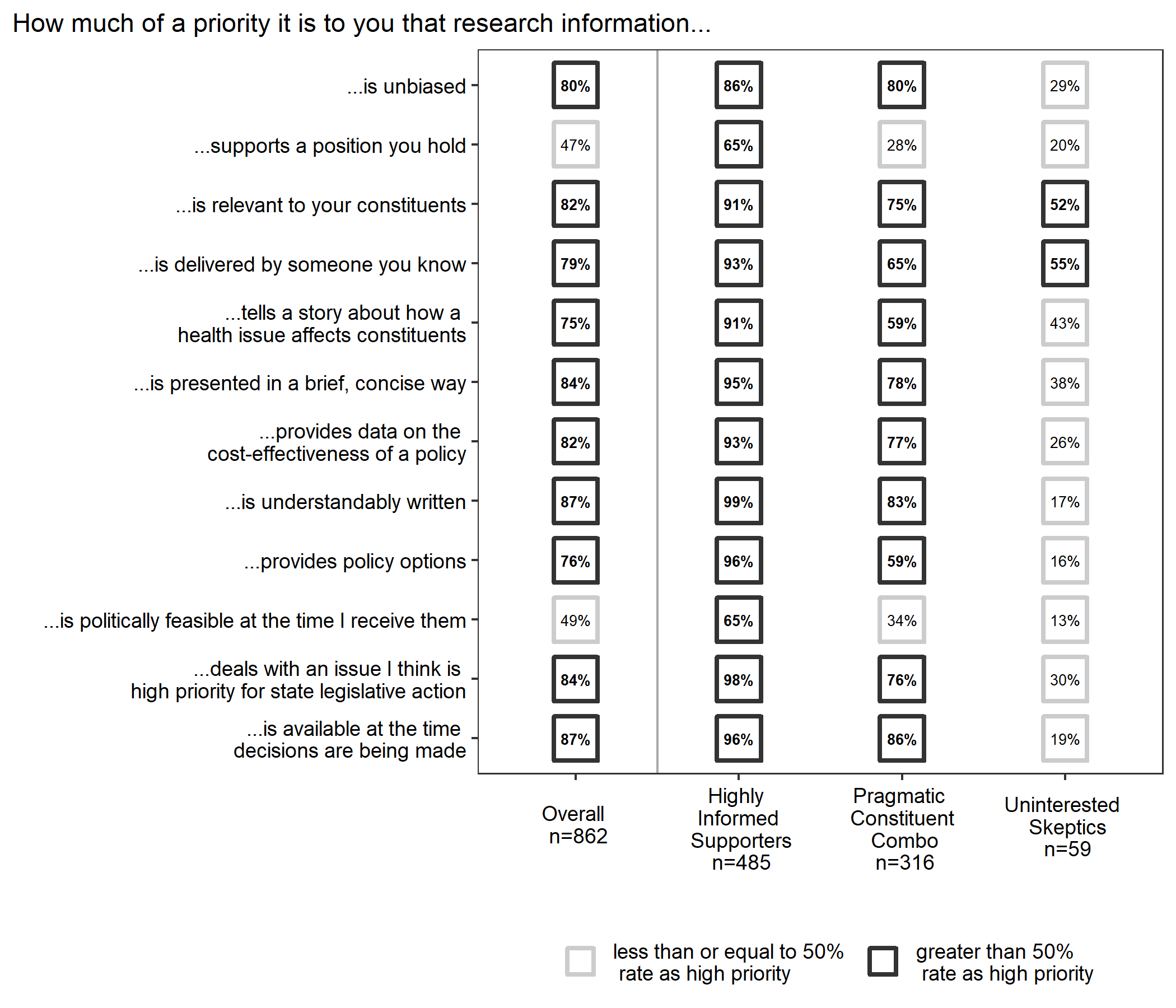


## Descriptive Statistics

### Table S6: Demographic and political variables

|  | Highly   Informed   Supporters (N=485) | Pragmatic   Constituent   Combo (N=316) | Uninterested   Skeptics (N=59) | Overall (N=862) |
| --- | --- | --- | --- | --- |
| **Years in legislature** |  |  |  |  |
| Mean (SD) | 9.54 (7.81) | 8.67 (8.03) | 8.32 (7.82) | 9.13 (7.89) |
| Median [Min, Max] | 8.00 [0, 40.0] | 6.00 [0, 56.0] | 6.00 [0, 32.0] | 6.00 [0, 56.0] |
| **Gender** |  |  |  |  |
| Female | 112 (23.1%) | 90 (28.5%) | 17 (28.8%) | 220 (25.5%) |
| Male | 373 (76.9%) | 226 (71.5%) | 42 (71.2%) | 642 (74.5%) |
| **Political Party** |  |  |  |  |
| Democrat | 233 (48.0%) | 131 (41.5%) | 26 (44.1%) | 392 (45.5%) |
| Republican | 242 (49.9%) | 180 (57.0%) | 31 (52.5%) | 453 (52.6%) |
| Other | 9 (1.9%) | 4 (1.3%) | 1 (1.7%) | 14 (1.6%) |
| Missing | 1 (0.2%) | 1 (0.3%) | 1 (1.7%) | 3 (0.3%) |
| **Reported ever sponsoring a health-related bill** | | | | |
| Yes | 314 (64.7%) | 189 (59.8%) | 34 (57.6%) | 539 (62.5%) |
| No | 161 (33.2%) | 117 (37.0%) | 23 (39.0%) | 301 (34.9%) |
| Don't know/Refused | 10 (2.1%) | 10 (3.2%) | 2 (3.4%) | 22 (2.6%) |
| **Census Region** |  |  |  |  |
| Northeast | 110 (22.7%) | 79 (25.0%) | 13 (22.0%) | 203 (23.5%) |
| Midwest | 120 (24.7%) | 86 (27.2%) | 13 (22.0%) | 219 (25.4%) |
| South | 165 (34.0%) | 97 (30.7%) | 17 (28.8%) | 280 (32.5%) |
| West | 87 (17.9%) | 54 (17.1%) | 14 (23.7%) | 155 (18.0%) |
| Territories or Puerto Rico | 3 (0.6%) | 0 (0%) | 2 (3.4%) | 5 (0.6%) |
| **Educational Attainment** |  |  |  |  |
| Less than college | 84 (17.3%) | 64 (20.3%) | 17 (28.8%) | 165 (19.1%) |
| College | 188 (38.8%) | 97 (30.7%) | 24 (40.7%) | 309 (35.8%) |
| More than college | 213 (43.9%) | 154 (48.7%) | 16 (27.1%) | 385 (44.7%) |
| Don't know/Refused | 0 (0%) | 1 (0.3%) | 2 (3.4%) | 3 (0.3%) |
| **Self-rated stance on social issues** | | | | |
| Liberal | 149 (30.7%) | 80 (25.3%) | 9 (15.3%) | 239 (27.7%) |
| Moderate | 100 (20.6%) | 54 (17.1%) | 16 (27.1%) | 171 (19.8%) |
| Conservative | 225 (46.4%) | 174 (55.1%) | 31 (52.5%) | 430 (49.9%) |
| Other | 2 (0.4%) | 1 (0.3%) | 1 (1.7%) | 4 (0.5%) |
| Don't know/Refused | 9 (1.9%) | 7 (2.2%) | 2 (3.4%) | 18 (2.1%) |
| **Self-rated stance on fiscal issues** | | | | |
| Liberal | 61 (12.6%) | 24 (7.6%) | 4 (6.8%) | 89 (10.3%) |
| Moderate | 105 (21.6%) | 55 (17.4%) | 15 (25.4%) | 175 (20.3%) |
| Conservative | 314 (64.7%) | 230 (72.8%) | 39 (66.1%) | 585 (67.9%) |
| Other | 1 (0.2%) | 1 (0.3%) | 0 (0%) | 2 (0.2%) |
| Don't know/Refused | 4 (0.8%) | 6 (1.9%) | 1 (1.7%) | 11 (1.3%) |
| **Self-rated health** |  |  |  |  |
| Excellent | 135 (27.8%) | 90 (28.5%) | 17 (28.8%) | 243 (28.2%) |
| Not Excellent | 350 (72.2%) | 225 (71.2%) | 40 (67.8%) | 616 (71.5%) |
| Don't know/Refused | 0 (0%) | 1 (0.3%) | 2 (3.4%) | 3 (0.3%) |
| **How often do you actively seek out research information when working on new policies?** | | | | |
| Never | 0 (0%) | 0 (0%) | 1 (1.7%) | 1 (0.1%) |
| Rarely | 11 (2.3%) | 2 (0.6%) | 2 (3.4%) | 15 (1.7%) |
| Sometimes | 78 (16.1%) | 53 (16.8%) | 18 (30.5%) | 149 (17.3%) |
| Most of the time | 232 (47.8%) | 169 (53.5%) | 23 (39.0%) | 425 (49.3%) |
| Always | 164 (33.8%) | 91 (28.8%) | 15 (25.4%) | 271 (31.4%) |
| Don't know/Refused | 0 (0%) | 1 (0.3%) | 0 (0%) | 1 (0.1%) |

### Table S7: Two of seven factors that most help to determine which health issues you work on

Specific question wording: “I’d like to know how important the following factors are in determining what health issues you work on. I will read you a list and ask that you tell me the top two factors.”

| Two factors that most help determine which health issues you work on | Highly   Informed   Supporters (N=485) | Pragmatic   Constituent   Combo (N=316) | Uninterested   Skeptics (N=59) | Overall (N=862) |
| --- | --- | --- | --- | --- |
| **Legislation being proposed by your colleagues** | | | | |
| Yes | 142 (29.3%) | 102 (32.3%) | 20 (33.9%) | 264 (30.6%) |
| No | 335 (69.1%) | 205 (64.9%) | 38 (64.4%) | 580 (67.3%) |
| Don't know/Refused | 8 (1.6%) | 9 (2.8%) | 1 (1.7%) | 18 (2.1%) |
| **Personal interest** | | | | |
| Yes | 119 (24.5%) | 61 (19.3%) | 9 (15.3%) | 189 (21.9%) |
| No | 358 (73.8%) | 246 (77.8%) | 49 (83.1%) | 655 (76.0%) |
| Don't know/Refused | 8 (1.6%) | 9 (2.8%) | 1 (1.7%) | 18 (2.1%) |
| **Research information** | | | | |
| Yes | 108 (22.3%) | 65 (20.6%) | 12 (20.3%) | 185 (21.5%) |
| No | 369 (76.1%) | 242 (76.6%) | 46 (78.0%) | 659 (76.5%) |
| Don't know/Refused | 8 (1.6%) | 9 (2.8%) | 1 (1.7%) | 18 (2.1%) |
| **Constituents' needs and opinions** | | | | |
| Yes | 323 (66.6%) | 200 (63.3%) | 46 (78.0%) | 571 (66.2%) |
| No | 154 (31.8%) | 107 (33.9%) | 12 (20.3%) | 273 (31.7%) |
| Don't know/Refused | 8 (1.6%) | 9 (2.8%) | 1 (1.7%) | 18 (2.1%) |
| **Data impacting your local area** | | | | |
| Yes | 132 (27.2%) | 97 (30.7%) | 16 (27.1%) | 245 (28.4%) |
| No | 345 (71.1%) | 210 (66.5%) | 42 (71.2%) | 599 (69.5%) |
| Don't know/Refused | 8 (1.6%) | 9 (2.8%) | 1 (1.7%) | 18 (2.1%) |
| **Interaction with lobbyists** | | | | |
| Yes | 41 (8.5%) | 18 (5.7%) | 4 (6.8%) | 64 (7.4%) |
| No | 436 (89.9%) | 289 (91.5%) | 54 (91.5%) | 780 (90.5%) |
| Don't know/Refused | 8 (1.6%) | 9 (2.8%) | 1 (1.7%) | 18 (2.1%) |
| **Economic issues** |  |  |  |  |
| Yes | 78 (16.1%) | 66 (20.9%) | 9 (15.3%) | 154 (17.9%) |
| No | 399 (82.3%) | 241 (76.3%) | 49 (83.1%) | 690 (80.0%) |
| Don't know/Refused | | | | |

### Table S8: Use of specific sources when making policy decisions

Specific question wording: “We know that policymakers must take a number of factors into account when making policy decisions. Now I’d like to ask you a few questions about how you use research information when you are working on an issue. I will read a series of statements and ask that you tell me, on a scale of 1 to 5, how often you use these things when you are making policy decisions. One means you never use them and 5 means you always use them. How often do you:”

| When making policy decisions, how often do you... | Highly   Informed   Supporters (N=485) | Pragmatic   Constituent   Combo (N=316) | Uninterested   Skeptics (N=59) | Overall (N=862) |
| --- | --- | --- | --- | --- |
| **...explore what other states are doing on the issue** | | | | |
| Never | 8 (1.6%) | 6 (1.9%) | 4 (6.8%) | 18 (2.1%) |
| 2 | 33 (6.8%) | 40 (12.7%) | 6 (10.2%) | 79 (9.2%) |
| 3 | 145 (29.9%) | 120 (38.0%) | 22 (37.3%) | 287 (33.3%) |
| 4 | 182 (37.5%) | 100 (31.6%) | 17 (28.8%) | 299 (34.7%) |
| Always | 116 (23.9%) | 50 (15.8%) | 10 (16.9%) | 178 (20.6%) |
| Not sure/Refused | 1 (0.2%) | 0 (0%) | 0 (0%) | 1 (0.1%) |
| **...read scientific research reports on the issue** | | | | |
| Never | 34 (7.0%) | 21 (6.6%) | 6 (10.2%) | 61 (7.1%) |
| 2 | 91 (18.8%) | 63 (19.9%) | 13 (22.0%) | 167 (19.4%) |
| 3 | 122 (25.2%) | 85 (26.9%) | 20 (33.9%) | 227 (26.3%) |
| 4 | 148 (30.5%) | 102 (32.3%) | 16 (27.1%) | 266 (30.9%) |
| Always | 88 (18.1%) | 44 (13.9%) | 4 (6.8%) | 138 (16.0%) |
| Not sure/Refused | 2 (0.4%) | 1 (0.3%) | 0 (0%) | 3 (0.3%) |
| **...read or watch popular media stories on the issue** | | | | |
| Never | 53 (10.9%) | 66 (20.9%) | 13 (22.0%) | 132 (15.3%) |
| 2 | 117 (24.1%) | 96 (30.4%) | 13 (22.0%) | 228 (26.5%) |
| 3 | 151 (31.1%) | 83 (26.3%) | 15 (25.4%) | 249 (28.9%) |
| 4 | 117 (24.1%) | 45 (14.2%) | 12 (20.3%) | 174 (20.2%) |
| Always | 45 (9.3%) | 24 (7.6%) | 5 (8.5%) | 74 (8.6%) |
| Not sure/Refused | 2 (0.4%) | 2 (0.6%) | 1 (1.7%) | 5 (0.6%) |
| **...ask internal legislative research bureaus for information on the issue** | | | | |
| Never | 12 (2.5%) | 6 (1.9%) | 5 (8.5%) | 23 (2.7%) |
| 2 | 19 (3.9%) | 24 (7.6%) | 5 (8.5%) | 48 (5.6%) |
| 3 | 69 (14.2%) | 50 (15.8%) | 17 (28.8%) | 136 (15.8%) |
| 4 | 152 (31.3%) | 110 (34.8%) | 20 (33.9%) | 283 (32.8%) |
| Always | 232 (47.8%) | 123 (38.9%) | 11 (18.6%) | 367 (42.6%) |
| Not sure/Refused | 1 (0.2%) | 3 (0.9%) | 1 (1.7%) | 5 (0.6%) |
| **...ask external legislative research organization for information on the issue** | | | | |
| Never | 29 (6.0%) | 28 (8.9%) | 4 (6.8%) | 62 (7.2%) |
| 2 | 65 (13.4%) | 54 (17.1%) | 18 (30.5%) | 138 (16.0%) |
| 3 | 120 (24.7%) | 111 (35.1%) | 18 (30.5%) | 249 (28.9%) |
| 4 | 173 (35.7%) | 82 (25.9%) | 16 (27.1%) | 271 (31.4%) |
| Always | 96 (19.8%) | 39 (12.3%) | 3 (5.1%) | 138 (16.0%) |
| Not sure/Refused | 2 (0.4%) | 2 (0.6%) | 0 (0%) | 4 (0.5%) |
| **...attend seminars or presentations where research is discussed** | | | | |
| Never | 48 (9.9%) | 51 (16.1%) | 11 (18.6%) | 110 (12.8%) |
| 2 | 127 (26.2%) | 91 (28.8%) | 18 (30.5%) | 236 (27.4%) |
| 3 | 151 (31.1%) | 108 (34.2%) | 17 (28.8%) | 278 (32.3%) |
| 4 | 124 (25.6%) | 46 (14.6%) | 10 (16.9%) | 180 (20.9%) |
| Always | 34 (7.0%) | 19 (6.0%) | 3 (5.1%) | 56 (6.5%) |
| Not sure/Refused | 1 (0.2%) | 1 (0.3%) | 0 (0%) | 2 (0.2%) |
| **...contact scientific researchers or experts for advice** | | | | |
| Never | 52 (10.7%) | 51 (16.1%) | 12 (20.3%) | 115 (13.3%) |
| 2 | 127 (26.2%) | 72 (22.8%) | 15 (25.4%) | 215 (24.9%) |
| 3 | 140 (28.9%) | 94 (29.7%) | 18 (30.5%) | 252 (29.2%) |
| 4 | 110 (22.7%) | 66 (20.9%) | 13 (22.0%) | 189 (21.9%) |
| Always | 54 (11.1%) | 29 (9.2%) | 1 (1.7%) | 84 (9.7%) |
| Not sure/Refused | 2 (0.4%) | 4 (1.3%) | 0 (0%) | 7 (0.8%) |
| **...take the results of a relevant scientific study into account when making a decision** | | | | |
| Never | 7 (1.4%) | 6 (1.9%) | 3 (5.1%) | 16 (1.9%) |
| 2 | 19 (3.9%) | 19 (6.0%) | 8 (13.6%) | 46 (5.3%) |
| 3 | 67 (13.8%) | 70 (22.2%) | 15 (25.4%) | 152 (17.6%) |
| 4 | 223 (46.0%) | 132 (41.8%) | 25 (42.4%) | 380 (44.1%) |
| Always | 166 (34.2%) | 85 (26.9%) | 7 (11.9%) | 260 (30.2%) |
| Not sure/Refused | 3 (0.6%) | 4 (1.3%) | 1 (1.7%) | 8 (0.9%) |
| **...talk with your colleagues about research on issues important to you** | | | | |
| Never | 4 (0.8%) | 3 (0.9%) | 1 (1.7%) | 8 (0.9%) |
| 2 | 19 (3.9%) | 18 (5.7%) | 7 (11.9%) | 44 (5.1%) |
| 3 | 63 (13.0%) | 71 (22.5%) | 13 (22.0%) | 148 (17.2%) |
| 4 | 158 (32.6%) | 104 (32.9%) | 23 (39.0%) | 286 (33.2%) |
| Always | 241 (49.7%) | 118 (37.3%) | 15 (25.4%) | 374 (43.4%) |
| Not sure/Refused | 0 (0%) | 2 (0.6%) | 0 (0%) | 2 (0.2%) |
| **...use research to justify a decision you made** | | | | |
| Never | 8 (1.6%) | 9 (2.8%) | 1 (1.7%) | 18 (2.1%) |
| 2 | 11 (2.3%) | 14 (4.4%) | 4 (6.8%) | 29 (3.4%) |
| 3 | 52 (10.7%) | 51 (16.1%) | 18 (30.5%) | 121 (14.0%) |
| 4 | 201 (41.4%) | 135 (42.7%) | 22 (37.3%) | 358 (41.5%) |
| Always | 211 (43.5%) | 101 (32.0%) | 14 (23.7%) | 328 (38.1%) |
| Not sure/Refused | 2 (0.4%) | 6 (1.9%) | 0 (0%) | 8 (0.9%) |
| **...use research presented in committee testimony** | | | | |
| Never | 4 (0.8%) | 6 (1.9%) | 1 (1.7%) | 11 (1.3%) |
| 2 | 16 (3.3%) | 19 (6.0%) | 5 (8.5%) | 41 (4.8%) |
| 3 | 61 (12.6%) | 66 (20.9%) | 13 (22.0%) | 140 (16.2%) |
| 4 | 176 (36.3%) | 118 (37.3%) | 23 (39.0%) | 317 (36.8%) |
| Always | 225 (46.4%) | 104 (32.9%) | 16 (27.1%) | 346 (40.1%) |
| Not sure/Refused | 3 (0.6%) | 3 (0.9%) | 1 (1.7%) | 7 (0.8%) |

### Table S9: Reliability of research information

Specific question wording: “Now I would like to know how reliable you believe research information is when it comes from different sources. On a scale of 1 to 5 with 1 meaning very unreliable and 5 meaning very reliable, please tell me how important it is to you that research information comes from:”

| How reliable research information is to you when it comes from ... | Highly   Informed   Supporters (N=485) | Pragmatic   Constituent   Combo (N=316) | Uninterested   Skeptics (N=59) | Overall (N=862) |
| --- | --- | --- | --- | --- |
| **... A university** |  |  |  |  |
| Very unreliable | 0 (0%) | 6 (1.9%) | 1 (1.7%) | 7 (0.8%) |
| 2 | 19 (3.9%) | 13 (4.1%) | 5 (8.5%) | 37 (4.3%) |
| 3 | 111 (22.9%) | 94 (29.7%) | 18 (30.5%) | 223 (25.9%) |
| 4 | 199 (41.0%) | 145 (45.9%) | 25 (42.4%) | 371 (43.0%) |
| Very reliable | 152 (31.3%) | 52 (16.5%) | 8 (13.6%) | 212 (24.6%) |
| Not sure/Refused | 4 (0.8%) | 6 (1.9%) | 2 (3.4%) | 12 (1.4%) |
| **... A government source** |  |  |  |  |
| Very unreliable | 15 (3.1%) | 13 (4.1%) | 8 (13.6%) | 36 (4.2%) |
| 2 | 44 (9.1%) | 52 (16.5%) | 12 (20.3%) | 109 (12.6%) |
| 3 | 193 (39.8%) | 146 (46.2%) | 21 (35.6%) | 360 (41.8%) |
| 4 | 187 (38.6%) | 83 (26.3%) | 16 (27.1%) | 287 (33.3%) |
| Very reliable | 37 (7.6%) | 16 (5.1%) | 1 (1.7%) | 54 (6.3%) |
| Not sure/Refused | 9 (1.9%) | 6 (1.9%) | 1 (1.7%) | 16 (1.9%) |
| **... Industry** |  |  |  |  |
| Very unreliable | 14 (2.9%) | 10 (3.2%) | 3 (5.1%) | 28 (3.2%) |
| 2 | 75 (15.5%) | 69 (21.8%) | 15 (25.4%) | 159 (18.4%) |
| 3 | 223 (46.0%) | 136 (43.0%) | 25 (42.4%) | 385 (44.7%) |
| 4 | 143 (29.5%) | 79 (25.0%) | 15 (25.4%) | 237 (27.5%) |
| Very reliable | 27 (5.6%) | 17 (5.4%) | 1 (1.7%) | 45 (5.2%) |
| Not sure/Refused | 3 (0.6%) | 5 (1.6%) | 0 (0%) | 8 (0.9%) |
| **... Advocacy groups** |  |  |  |  |
| Very unreliable | 24 (4.9%) | 32 (10.1%) | 11 (18.6%) | 67 (7.8%) |
| 2 | 92 (19.0%) | 95 (30.1%) | 19 (32.2%) | 208 (24.1%) |
| 3 | 245 (50.5%) | 144 (45.6%) | 19 (32.2%) | 408 (47.3%) |
| 4 | 105 (21.6%) | 33 (10.4%) | 9 (15.3%) | 147 (17.1%) |
| Very reliable | 11 (2.3%) | 4 (1.3%) | 1 (1.7%) | 16 (1.9%) |
| Not sure/Refused | 8 (1.6%) | 8 (2.5%) | 0 (0%) | 16 (1.9%) |
| **... Constituents** |  |  |  |  |
| Very unreliable | 6 (1.2%) | 9 (2.8%) | 4 (6.8%) | 20 (2.3%) |
| 2 | 44 (9.1%) | 34 (10.8%) | 9 (15.3%) | 87 (10.1%) |
| 3 | 174 (35.9%) | 136 (43.0%) | 28 (47.5%) | 338 (39.2%) |
| 4 | 181 (37.3%) | 94 (29.7%) | 13 (22.0%) | 288 (33.4%) |
| Very reliable | 74 (15.3%) | 36 (11.4%) | 5 (8.5%) | 115 (13.3%) |
| Not sure/Refused | 6 (1.2%) | 7 (2.2%) | 0 (0%) | 14 (1.6%) |
| **... The media** |  |  |  |  |
| Very unreliable | 89 (18.4%) | 100 (31.6%) | 21 (35.6%) | 212 (24.6%) |
| 2 | 174 (35.9%) | 134 (42.4%) | 23 (39.0%) | 331 (38.4%) |
| 3 | 187 (38.6%) | 69 (21.8%) | 14 (23.7%) | 270 (31.3%) |
| 4 | 30 (6.2%) | 8 (2.5%) | 1 (1.7%) | 39 (4.5%) |
| Very reliable | 1 (0.2%) | 0 (0%) | 0 (0%) | 1 (0.1%) |
| Not sure/Refused | 4 (0.8%) | 5 (1.6%) | 0 (0%) | 9 (1.0%) |
| **... Other legislators** |  |  |  |  |
| Very unreliable | 7 (1.4%) | 5 (1.6%) | 0 (0%) | 12 (1.4%) |
| 2 | 35 (7.2%) | 36 (11.4%) | 7 (11.9%) | 79 (9.2%) |
| 3 | 209 (43.1%) | 151 (47.8%) | 37 (62.7%) | 397 (46.1%) |
| 4 | 194 (40.0%) | 104 (32.9%) | 13 (22.0%) | 312 (36.2%) |
| Very reliable | 34 (7.0%) | 12 (3.8%) | 2 (3.4%) | 48 (5.6%) |
| Not sure/Refused | 6 (1.2%) | 8 (2.5%) | 0 (0%) | 14 (1.6%) |
| **... Caucus leadership** |  |  |  |  |
| Very unreliable | 28 (5.8%) | 29 (9.2%) | 3 (5.1%) | 60 (7.0%) |
| 2 | 56 (11.5%) | 52 (16.5%) | 14 (23.7%) | 123 (14.3%) |
| 3 | 178 (36.7%) | 123 (38.9%) | 25 (42.4%) | 326 (37.8%) |
| 4 | 179 (36.9%) | 86 (27.2%) | 13 (22.0%) | 279 (32.4%) |
| Very reliable | 32 (6.6%) | 17 (5.4%) | 4 (6.8%) | 53 (6.1%) |
| Not sure/Refused | 12 (2.5%) | 9 (2.8%) | 0 (0%) | 21 (2.4%) |

# Survey Instrument

**Telephone Interview Script**

Thank you for agreeing to participate in this interview. As you know, we are interested in learning about your thoughts on and use of different types of information in your work. Specifically, we’d like to know where you go for information, how you prefer to receive it, and a little bit about your policy priorities.

We would like to have your permission to tape this interview. Any information we gather will be kept confidential and will not be attributed to any individual. Do I have your permission to record this interview? [Note: if they refuse, proceed with interview unrecorded.]

1. I’d like to know how important the following factors are in determining what health issues you work on. I will read you a list and ask that you tell me the top two factors.
   1. Legislation being proposed by your colleagues
   2. Personal interest
   3. Research information
   4. Constituents’ needs and opinions
   5. Data on impact in my local area
   6. Interaction with lobbyists
   7. Economic issues
2. What percent of what you hope to accomplish during the legislative session have you already worked on before the session begins?
3. Now I would like to ask you a few questions about the issues most important to you. Some legislators select an issue or two where they most want to make a difference. What issues are your legislative priorities?
4. In your opinion, what would you say are the top three, most important health issues for policy action in your state?

*Prompts, if needed:*

| Obesity | Cancer | Tobacco use/cessation |
| --- | --- | --- |
| Medicare/Medicaid | HIV/AIDS | Heart disease |
| Diabetes | Violence prevention | Injury prevention |
| Mental health | The environment | Aging |
| Infectious diseases | Physical activity | Diet/nutrition |
| Access to healthcare | Quality of healthcare | Universal coverage |
| Prescription drug abuse | Other__________________ |  |

1. Do you work on any issues related to cancer?

If yes, which ones? Why have you chosen to work on that issue?

1. What could make research information more useful to you when you work on health issues?
2. We know that policymakers must take a number of factors into account when making policy decisions. Now I’d like to ask you a few questions about how you use research information when you are working on an issue. I will read a series of statements and ask that you tell me, on a scale of 1 to 5, how often you use these things when you are making policy decisions. One means you *never* use them and 5 means you *always* use them. How often do you:

| Explore what other states are doing on the issue | 1 | 2 | 3 | 4 | 5 | DK/ref |
| --- | --- | --- | --- | --- | --- | --- |
| Read scientific research reports on the issue | 1 | 2 | 3 | 4 | 5 | DK/ref |
| Read or watch popular media stories on the issue | 1 | 2 | 3 | 4 | 5 | DK/ref |
| Ask internal legislative research bureaus for information on the issue | 1 | 2 | 3 | 4 | 5 | DK/ref |
| Ask an external legislative research organization for information on the issue | 1 | 2 | 3 | 4 | 5 | DK/ref |
| Attend seminars or presentations where research is discussed | 1 | 2 | 3 | 4 | 5 | DK/ref |
| Contact scientific researchers or experts for advice | 1 | 2 | 3 | 4 | 5 | DK/ref |
| Take the results of a relevant scientific study into account when making a decision | 1 | 2 | 3 | 4 | 5 | DK/ref |
| Talk with your colleagues about research on issues important to you | 1 | 2 | 3 | 4 | 5 | DK/ref |
| Use research to justify a decision you made | 1 | 2 | 3 | 4 | 5 | DK/ref |
| Use research presented in committee testimony | 1 | 2 | 3 | 4 | 5 | DK/ref |

1. We know you spend a lot of time hearing testimony in committee hearings. How does this testimony influence your decisions about policy?
2. What about the testimony affects its influence on you?

Prompts: Who delivers the testimony, the timing of the testimony, the method or format of the testimony delivery, the use of visual aids, etc.

1. Now I’d like to learn a little about what makes research information useful to you. I am going to read a series of statements about research information and I would like you to tell me how much of a priority it is to you that research information has various characteristics. You can answer on a scale of 1 to 5, with 1 meaning *low priority* and 5 meaning *high priority*.

| **Source** | | | | | | |
| --- | --- | --- | --- | --- | --- | --- |
|  | | | | | | |
| Research information is unbiased | 1 | 2 | 3 | 4 | 5 | DK/ref |
| Research information supports a position I hold | 1 | 2 | 3 | 4 | 5 | DK/ref |
| Research information is relevant to my constituents | 1 | 2 | 3 | 4 | 5 | DK/ref |
| Research information is delivered to me by someone I know or respect | 1 | 2 | 3 | 4 | 5 | DK/ref |
| **Presentation** | | | | | | |
| Research information tells a story of how a health issue affects my constituents | 1 | 2 | 3 | 4 | 5 | DK/ref |
| Research information is presented in a brief, concise way | 1 | 2 | 3 | 4 | 5 | DK/ref |
| Research information provides data on the cost-effectiveness of a policy | 1 | 2 | 3 | 4 | 5 | DK/ref |
| Research information is understandably written | 1 | 2 | 3 | 4 | 5 | DK/ref |
| Research information provides policy options | 1 | 2 | 3 | 4 | 5 | DK/ref |
| **Timeliness** | | | | | | |
| Research implications are politically feasible at the time I receive them | 1 | 2 | 3 | 4 | 5 | DK/ref |
| Research information deals with an issue that I think is a high priority for state legislative policy action | 1 | 2 | 3 | 4 | 5 | DK/ref |
| Research information is available at the time decisions are being made | 1 | 2 | 3 | 4 | 5 | DK/ref |

1. Now I would like to know how reliable you believe research information is when it comes from different sources. On a scale of 1 to 5 with 1 meaning *very unreliable* and 5 meaning *very reliable*, please tell me how important it is to you that research information comes from:

| A university | 1 | 2 | 3 | 4 | 5 | DK/ref |
| --- | --- | --- | --- | --- | --- | --- |
| A government source | 1 | 2 | 3 | 4 | 5 | DK/ref |
| Industry | 1 | 2 | 3 | 4 | 5 | DK/ref |
| Advocacy groups | 1 | 2 | 3 | 4 | 5 | DK/ref |
| Constituents | 1 | 2 | 3 | 4 | 5 | DK/ref |
| The media | 1 | 2 | 3 | 4 | 5 | DK/ref |
| Other legislators | 1 | 2 | 3 | 4 | 5 | DK/ref |
| Caucus leadership | 1 | 2 | 3 | 4 | 5 | DK/ref |

1. On a scale from 1 to 5 with 1 meaning *never* and 5 meaning *always*, how often do you actively seek out research information when working on new policies?
2. If you seek research information to inform your policy decisions, who can you count on to quickly give you what you need?

Now I just have a few final questions about you.

1. Have you, your spouse, a first-degree relative of yours (parents, children, brother, sister) ever been diagnosed with cancer?
2. Do you have any children?
3. When it comes to SOCIAL issues do you usually think of yourself as
   1. Extremely liberal
   2. Liberal
   3. Slightly liberal
   4. Moderate
   5. Slightly conservative
   6. Conservative
   7. Extremely conservative
   8. Other
   9. Don’t know/refused
4. When it comes to FISCAL issues do you usually think of yourself as:
5. Extremely liberal
6. Liberal
7. Slightly liberal
8. Moderate
9. Slightly conservative
10. Conservative
11. Extremely conservative
12. Other
13. Don’t know/refused
14. Would you say that in general your health is:
15. Excellent
16. Very good
17. Good
18. Fair
19. Poor
20. Don’t know/refused
21. What is the highest level of education that you have completed?
22. Some high school or less
23. High school graduate
24. Trade, technical, or vocational education beyond high school
25. Some college
26. College degree
27. Postgraduate degree
28. For how many years have you served in the legislature?
29. Have you ever sponsored a bill about a health topic?
30. If yes, go to question 9.
31. If no, go to question 10.
32. How long ago did you sponsor a bill about a health topic?
33. In the last session
34. 2-5 years ago
35. More than 5 years ago
36. Is there anything else you’d like to add about anything we’ve talked about?

Thank you very much for your help. If you are interested in the findings from our project, we are glad to provide this information to you when they are available. (Get contact info if interested.)

If you have any questions you may contact Beth Dodson by phone at 314-362-9652 or by email at [edodson@wustl.edu](mailto:edodson@wustl.edu). If you were unhappy with your experience or wish to express a complaint, please contact Beth Dodson or the Project Director, Dr. Ross Brownson, at 314-362-9643. If you would like to speak with someone about your rights as a research participant, please call the Human Research Protection Office at 314-633-7400 or 800-438-0445. The Human Research Protection team is comprised of employees of Washington University who are not part of the research team. Their jobs are to make sure that research participants’ rights are protected.
